# Favorable outcome of immunotherapy use in metastatic HR+/HER2− breast cancer: a population-based cohort study

**DOI:** 10.1097/JS9.0000000000003556

**Published:** 2025-09-23

**Authors:** Guansheng Zhong, Jingjing Hu, Luxin Wei, Bajin Wei, Haidong Cui, Weiyang Lou, Jinfei Ma, Wanting Shao, Lifeng Zhang, Zhijun Dai

**Affiliations:** a Department of Breast Surgery, College of Medicine, The First Affiliated Hospital, Zhejiang University, Hangzhou, Zhejiang, China; b Massachusetts General Cancer Center, Boston, MA, USA; c Department of General Surgery, The Fourth Affiliated Hospital of Soochow University (Suzhou Dushu Lake Hospital), Suzhou, Jiangsu, China

**Keywords:** breast cancer, HR+HER2−, metastasis, NCDB, survival

## Abstract

**Background:**

Uncertainty persists regarding the efficacy of immunotherapy for patients with advanced HR+/HER2− breast cancer. We aimed to assess the survival benefit of immunotherapy in metastatic HR+/HER2− breast cancer.

**Methods:**

Patients diagnosed with *de novo* metastatic HR+/HER2− breast cancer from 2013–2021 were analyzed using data from the National Cancer Database. Logistic regression analysis was performed to estimate factors linked to immunotherapy use. Survival outcomes were evaluated through propensity score-matching (PSM), along with Kaplan–Meier methodology and Cox regression models.

**Results:**

A total of 17 211 metastatic HR+/HER2− breast cancer patients were included, of whom 1571 patients receiving immunotherapy were matched 1:1 with 1571 patients not receiving immunotherapy. The immunotherapy use was more common among those with higher burden of lymph node metastasis [odds ratio (OR): 1.35; 95% confidence intervals (CIs): [1.13–1.62]; *P=* 0.001], and those received hormone therapy (OR: 1.21; 95% CI: [1.04–1.39]; *P =*0.012) and radiotherapy (OR: 1.19; 95% CI: [1.06–1.33]; *P=* 0.002). Survival analysis indicated that patients who had undergone immunotherapy had a significantly improved overall survival (OS) compared with patients who had not both before (hazard ratio [HR]: 0.75; 95% CI: [0.70–0.80]; *P* < 0.001) and after PSM (HR: 0.77; 95% CI: [0.70–0.85]; *P* < 0.001). Moreover, survival benefit from usage of immunotherapy was also statistically significant in subgroups received hormone therapy (HR: 0.62; 95% CI: [0.51–0.76]; *P* < 0.001) or chemotherapy (HR: 0.67; 95% CI: [0.48–0.92]; *P =* 0.015) only.

**Conclusion:**

Immunotherapy may be beneficial for the OS of patients with *de novo* metastatic HR+/HER2− breast cancer. Our data may provide clues for the integration of immunotherapy into future clinical treatment strategies.

**HIGHLIGHTS**

- This study thoroughly evaluated the survival benefit of immunotherapy in metastatic HR+/HER2− breast cancer.
- Our NCDB analysis suggests that immunotherapy provides a significant survival benefit for metastatic HR+/HER2− breast cancer.
- The survival benefit was maintained across subgroups receiving either hormone therapy or chemotherapy alone.

## Introduction

Metastatic HR+/HER2− breast cancer accounts for approximately 73% of all metastatic breast cancer cases^[1]^. The current standard treatments primarily include endocrine therapy, CDK4/6 inhibitors, and chemotherapy^[2]^. These therapies have significantly improved survival of patients, with the median OS extending to 2–5 years^[3]^. However, most patients ultimately experience disease progression and lack further effective treatment options. Consequently, new treatment strategies are urgently needed to improve survival in patients with advanced HR+/HER2− breast cancer.

Recently, immunotherapy, particularly immune checkpoint inhibitors (ICIs), has achieved significant breakthroughs in treating metastatic triple-negative breast cancer (mTNBC), substantially altering the treatment paradigm for this subtype^[4]^. In the IMpassion130 trial^[5]^, atezolizumab combined with nab-paclitaxel significantly extended progression-free survival (PFS) in programmed cell death 1 ligand 1 (PD-L1)-positive mTNBC patients, while in the KEYNOTE-355 trial^[6]^, pembrolizumab combined with various chemotherapy regimens significantly improved OS in PD-L1-high expressing mTNBC patients. These studies have led to the widespread application of immunotherapy in mTNBC, sparking interest in its potential application in HR+/HER2− breast cancer.

Although HR+/HER2− breast cancer typically exhibits low PD-L1 expression and low tumor-infiltrating lymphocytes (TILs) level, which are generally linked to limited response to immunotherapy, several exploratory studies suggest that some HR+/HER2− patients may still benefit from immunotherapy, either as monotherapy or in combination therapy^[4]^. For example, the KEYNOTE-028 study demonstrated modest antitumor activity with pembrolizumab monotherapy in metastatic HR+/HER2− breast cancer patients^[7]^. Similarly, JAVELIN Solid Tumor trial^[8]^ reported similar results with avelumab. Furthermore, the KELLY trial evaluated the combination of pembrolizumab and ibrutinib, showing positive antitumor activity in advanced HR+/HER2− breast cancer patients^[9]^. In the NCT02778685 trial, palbociclib in combination with letrozole and pembrolizumab as a first-line treatment showed a 31% complete response rate and good tolerability^[10]^. Additionally, research exploring the combination of immunotherapy with antibody-drug conjugates, such as Sacituzumab govitecan, which targets Trop-2 and releases a topoisomerase I inhibitor payload, has indicated encouraging results in enhancing the antitumor effects of immunotherapy^[11,12]^. Despite these studies being in early stages with small sample sizes and limited follow-up, they provide important insights into the potential application of immunotherapy in mHR+/HER2− breast cancer.

The National Cancer Database (NCDB) provides a powerful platform for analyzing real-world data from a large cohort of patients in the US^[13]^. By utilizing this data, we aim to study the clinical and sociodemographic factors influencing the application of immunotherapy in metastatic HR+/HER2− breast cancer and assess its impact on patient survival. Our study will offer valuable insights into the role of immunotherapy in advanced HR+/HER2− breast cancer and provide guidance for its integration into future clinical treatment strategies. Through this analysis, we hope to provide strong support for improving treatment plans for metastatic HR+/HER2− breast cancer patients and optimize future immunotherapy strategies to improve patient outcomes.

## Patients and methods

### Patient cohort and data source

We analyzed data from the NCDB, a comprehensive cancer registry that encompasses approximately 70% of newly diagnosed malignancies across the United States. Our analysis focused on individuals with an initial diagnosis of metastatic HR+/HER2− breast cancer between 2013 and 2021, specifically those who did not undergo surgical intervention for breast cancer.

**Figure 1.**
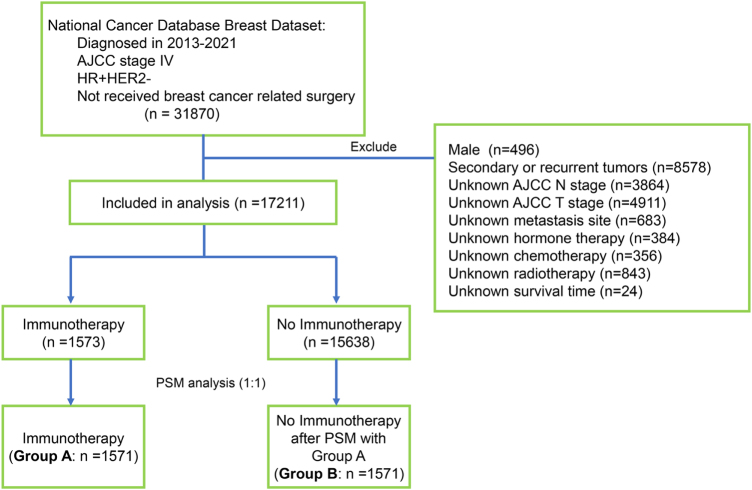
The schematic representation of patient selection.

Exclusion criteria applied in this investigation comprised: (1) secondary or recurrent tumors; (2) male breast cancer cases; (3) incomplete documentation of AJCC T and N staging, radiotherapy, cytotoxic therapy, hormone therapy, or survival times. Based on these criteria, a total of 17 211 eligible patients were included in the study, of whom 15 638 did not receive immunotherapy and 1573 received immunotherapy. Subsequently, 1571 patients receiving immunotherapy were matched with 1571 patients not receiving immunotherapy by using the propensity score matching with a ratio of 1:1. The detailed flowchart for the patients’ selection is shown in Figure 1. This cohort study has been reported in line with the STROCSS guidelines^[14]^.

### Propensity score-matching analysis

In order to reduce possible selection bias for survival comparison, a one-to-one PSM between patients with or without immunotherapy was conducted by utilizing the nearest-neighbor matching method with a caliper of 0.05. We included all the covariates in patients’ baseline characteristics into the propensity score-matching (PSM) algorithm, including age, race, income, location, diagnosis year, T and N stage, treatment modalities (endocrine therapy, chemotherapy, and radiotherapy), and metastasis sites (bone, brain, liver, and lung). A histogram of standardized differences before and after PSM was plotted to visually exhibit the properties of the matching procedure (Supplemental Digital Content Figure 1, available at: http://links.lww.com/JS9/F208). The PSM procedure and the calculation of standardized differences were performed using R packages of MatchIt.

### Statistical analysis

We used descriptive statistics to present all demographic and clinicopathological variables. Associations between clinicopathological parameters were examined using the Pearson χ^2^ test. For survival outcomes, we constructed Kaplan–Meier curves and Cox regression modeling to assess differences between groups. Variables with *P* < 0.10 on univariate analysis were included in the multivariate analysis. To identify variables that influenced immunotherapy administration, we conducted logistic regression analysis. Odds ratios (ORs) and hazard ratios (HRs) with corresponding 95% confidence intervals (CIs) were derived through logistic regression modeling and Cox regression modeling, respectively.

The statistical computations for descriptive analyses, Pearson χ^2^ testing, logistic regression, and Cox proportional hazards modeling were executed with SPSS software version 24.0 (IBM Corp). R software (version 4.2.2) was utilized to perform the Kaplan–Meier survival analyses. Statistical significance was established at a two-sided *P* value below 0.05.

## Results

### Baseline characteristics

A total of 17 211 metastatic HR+/HER2− breast cancer patients were included in our final analysis, with 1573 individuals (9.1%) having received immunotherapy and 15 638 (90.9%) not receiving it. Baseline characteristics were compared between groups using the Pearson χ^2^ test (Table 1). The age distribution among patients receiving immunotherapy skewed younger, with a higher proportion in the 35–50 (19.6% vs 15.9%, *P* < 0.001) age range, and a smaller percentage in the 70+ age group (27.7% vs 33.9%, *P* < 0.001). Immunotherapy use was more common among white patients (79.0% vs 76.8%, *P=* 0.030). Patients receiving immunotherapy had higher rates of advanced AJCC N stage (N3; 15.3% vs 13.3%, *P=* 0.012), bone (87.2% vs 77.9%, *P* < 0.001), and lung (24.3% vs 20.2%, *P* < 0.001) metastases. Additionally, patients receiving immunotherapy were more likely to receive hormone therapy (84.5% vs 80.2%, *P* < 0.001), chemotherapy (64.1% vs 59.8%, *P* < 0.001), and radiotherapy (33.6% vs 28.9%, *P* < 0.001).

**Table 1:** Characteristics of patients with *de novo* metastatic HR+HER2− breast cancer who have not received surgical intervention

Characteristic	No immunotherapy (*N* = 15 638) No. (%)	Immunotherapy (*N* = 1573) No. (%)	*P* value Before PSM	No immunotherapy (*N* = 1571) No. (%)	Immunotherapy (*N* = 1571) No. (%)	*P* value After PSM	SMD
	Before PSM			After PSM			
Age	–	–	<0.001	–	–	0.516	0.054
<35	386 (2.5)	44 (2.8)	–	44 (2.8)	42 (2.7)	–	–
35–50	2480 (15.9)	308 (19.6)	–	276 (17.6)	308 (19.6)	–	–
51–69	7478 (47.8)	785 (49.9)	–	796 (50.7)	785 (50.0)	–	–
≥ 70	5294 (33.9)	436 (27.7)	–	455 (29.0)	436 (27.8)	–
Race	–	–	0.030	–	–	0.331	0.053
White	12 015 (76.8)	1243 (79.0)	–	1267 (80.6)	1241 (79.0)	–	–
Black	2611 (16.7)	222 (14.1)	–	215 (13.7)	222 (14.1)	–	–
Others^a^/Unknown	1012 (6.5)	108 (6.9)	–	89 (5.7)	108 (6.9)	
Income	–	–	0.114	–	–	0.959	0.028
<$38 000	2524 (16.1)	220 (14.0)	–	231 (14.7)	220 (14.0)	–	–
$38 000–47 999	3029 (19.4)	292 (18.6)	–	286 (18.2)	292 (18.6)	–	–
$48 000–62 999	3572 (22.8)	389 (24.7)	–	386 (24.6)	388 (24.7)	–	–
$63 000 or more	4422 (28.3)	462 (29.4)	–	468 (29.8)	461 (29.3)	–	–
Unknown	2091 (13.4)	210 (13.4)	–	200 (12.7)	210 (13.4)	
Location	–	–	0.078	–	–	0.911	0.026
Metro	12 792 (81.8)	1324 (84.2)	–	1332 (84.8)	1322 (84.2)	–	–
Urban	2027 (13.0)	171 (10.9)	–	166 (10.6)	171 (10.9)	–	–
Rural	295 (1.9)	32 (2.0)	–	27 (1.7)	32 (2.0)	–	–
Unknown	524 (3.4)	46 (2.9)	–	46 (2.9)	46 (2.9)	
Diagnose year	–	–	0.083	–	–	0.971	0.001
2013–2017	6637 (42.4)	632 (40.2)		632 (40.2)	631 (40.2)	–	–
2018–2021	9001 (57.6)	941 (59.8)		939 (59.8)	940 (59.8)	
T stage	–	–	0.135	–	–	0.430	0.059
T1	2352 (15.0)	251 (16.0)	–	227 (14.4)	250 (15.9)	–	–
T2	5394 (34.5)	560 (35.6)	–	569 (36.2)	559 (35.6)	–	–
T3	2768 (17.7)	292 (18.6)	–	274 (17.4)	292 (18.6)	–	–
T4	5124 (32.8)	470 (29.9)	–	501 (31.9)	470 (29.9)	
N stage	–	–	0.012	–	–	0.856	0.031
N0	3810 (24.4)	347 (22.1)		353 (22.5)	347 (22.1)	–	–
N1	7905 (50.5)	823 (52.3)		828 (52.7)	822 (52.3)	–	–
N2	1841 (11.8)	162 (10.3)		167 (10.6)	162 (10.3)	–	–
N3	2082 (13.3)	241 (15.3)		223 (14.2)	240 (15.3)	
Hormone therapy	–	–	<0.001	–	–	0.454	0.027
No	3090 (19.8)	244 (15.5)		229 (14.6)	244 (15.5)	–	–
Yes	12 548 (80.2)	1329 (84.5)		1342 (85.4)	1327 (84.5)	
Chemotherapy	–	–	0.001	–	–	0.882	0.005
No	6282 (40.2)	565 (35.9)	–	569 (36.2)	565 (36.0)	–	–
Yes	9356 (59.8)	1008 (64.1)	–	1002 (63.8)	1006 (64.0)	
Radiotherapy	–	–	<0.001	–	–	0.649	0.016
No	11 117 (71.1)	1045 (66.4)		1056 (67.2)	1044 (66.5)	–	–
Yes	4521 (28.9)	528 (33.6)		515 (32.8)	527 (33.5)	
Metastatic site	–	–	–	–	–	–	–
Bone	12 189 (77.9)	1372 (87.2)	<0.001	1384 (88.1)	1370 (87.2)	0.448	0.027
Brain	975 (6.2)	103 (6.5)	0.625	88 (5.6)	103 (6.6)	0.263	0.040
Liver	3065 (19.6)	322 (20.5)	0.408	283 (18.0)	322 (20.5)	0.078	0.063
Lung	3159 (20.2)	382 (24.3)	<0.001	370 (23.6)	24.3 (24.3)	0.616	0.018

PSM, propensity score matching.SMD, standardized mean difference;

^a^ Other races included American Indians, Chinese, Japanese, Filipino, Hawaiian, Korean, Vietnamese, etc.

### Factors associated with immunotherapy use

To identify factors related to immunotherapy use, a multivariate logistic regression analysis was carried out (Table 2). Patients of the black race were less likely to receive immunotherapy compared to the white race (OR = 0.83; 95% CI: [0.71–0.97]; *P* = 0.022). Individuals residing in urban settings had a reduced probability of immunotherapy treatment relative to those in metropolitan regions (OR = 0.82; 95% CI: [0.69–0.97]; *P* = 0.022). In terms of clinical characteristics, later AJCC N stage (N3 vs N0: OR = 1.35; 95% CI: [1.13–1.62]; *P* = 0.001), bone (OR = 1.81; 95% CI: [1.54–2.11]; *P* < 0.001) and lung (OR = 1.15; 95% CI: [1.02–1.30]; *P* = 0.026) metastases was associated with increased odds of immunotherapy use. Moreover, treatment modalities emerged as significant predictors. Hormone therapy (OR = 1.21; 95% CI: [1.04–1.39]; *P* = 0.012) and radiotherapy (OR = 1.19; 95% CI: [1.06–1.33]; *P* = 0.002) displayed strong associations with immunotherapy use. However, despite showing correlation in univariate analysis, chemotherapy did not maintain statistical significance in the multivariate model (OR = 1.08; 95% CI: [0.96–1.21]; *P* = 0.184).

**Table 2:** Logistic regression analysis for factors associated with immunotherapy use in *de novo* metastatic HR+HER2− breast cancer who have not received surgical intervention

	Univariate analysis		Multivariate analysis	
Variables	OR (95% CI)	*P* value	OR (95% CI)	*P* value
Age		<0.001		<0.001
	Reference		Reference	
35–49	1.09 (0.78–1.52)	0.614	1.11 (0.80–1.56)	0.534
50–69	0.92 (0.67–1.27)	0.614	0.93 (0.68–1.29)	0.668
≥ 70	0.72 (0.52–1.00)	0.051	0.77 (0.55–1.07)	0.124
Race		0.030		0.072
White	Reference		Reference	
Black	0.82 (0.71–0.95)	0.010	0.83 (0.71–0.97)	0.022
Others/unknown	1.03 (0.84–1.27)	0.768	0.99 (0.80–1.22)	0.902
Income	–	0.114	–	0.485
<$38000	Reference		Reference	
$38 000–47 999	1.11 (0.92–1.33)	0.280	1.06 (0.88–1.28)	0.522
$48 000–62 999	1.25 (1.05–1.49)	0.012	1.18 (0.98–1.41)	0.077
$63 000 or more	1.20 (1.01–1.42)	0.034	1.10 (0.92–1.31)	0.296
Unknown	1.15 (0.95–1.40)	0.160	1.09 (0.89–1.33)	0.428
Location	–	0.079	–	0.073
Metro	Reference		Reference	
Urban	0.82 (0.69–0.96)	0.016	0.82 (0.69–0.97)	0.022
Rural	1.05 (0.73–1.52)	0.803	1.04 (0.72–1.51)	0.830
Unknown	0.85 (0.62–1.15)	0.293	0.81 (0.59–1.10)	0.167
Diagnose year	–	0.083	–	0.160
2013–2017	Reference			
2018–2021	1.10 (0.99–1.22)		1.08 (0.97–1.21)	0.160
T stage	–	0.135	–	0.105
T1	Reference		Reference	
T2	0.97 (0.83–1.14)	0.730	0.94 (0.80–1.10)	0.448
T3	0.99 (0.83–1.18)	0.898	0.94 (0.79–1.13)	0.521
T4	0.86 (0.73–1.01)	0.065	0.83 (0.70–0.98)	0.027
N stage	–	0.012	–	0.006
N0	Reference		Reference	
N1	1.14 (1.00–1.30)	0.046	1.16 (1.01–1.32)	0.034
N2	0.97 (0.80–1.17)	0.729	1.04 (0.85–1.28)	0.684
N3	1.27 (1.07–1.51)	0.007	1.35 (1.13–1.62)	0.001
Hormone therapy	–	<0.001	–	0.012
No	Reference		Reference	
Yes	1.34 (1.16–1.55)		1.21 (1.04–1.39)	
Chemotherapy	–	0.001	–	0.184
No	Reference		Reference	
Yes	1.20 (1.08–1.33)		1.08 (0.96–1.21)	
Radiotherapy	–	<0.001	–	0.002
No	Reference		Reference	
Yes	1.24 (1.11–1.39)		1.19 (1.06–1.33)	
Metastatic site	–	–	–	–
Bone	1.93 (1.66–2.25)	<0.001	1.81 (1.54–2.11)	<0.001
Brain	1.05 (0.85–1.30)	0.625	NA	
Liver	1.06 (0.93–1.20)	0.408	NA	
Lung	1.25 (1.11–1.41)	<0.001	1.15 (1.02–1.30)	0.026

CI, confidence interval; OR, odds ratio.

### Survival analyses

**Figure 2.**
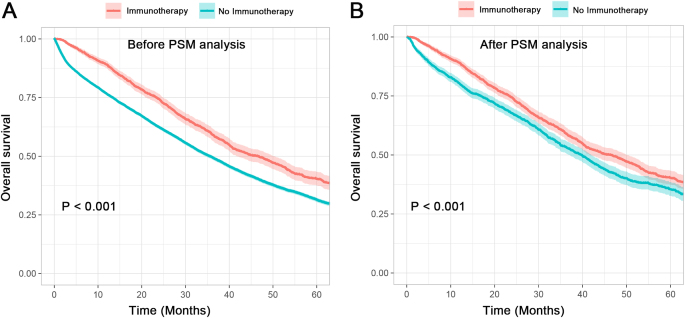
Overall survival of *de novo* metastatic HR+/HER2− breast cancer patients with or without immunotherapy. (A) Total cohort comparison and (B) post-PSM method comparison.

The overall survival (OS) of patients who had received immunotherapy or not was compared by the Kaplan–Meier method. As exhibited in Figure 2A, patients who had undergone immunotherapy had a superior OS compared with patients who had not (median 46.5 vs 35.5 months, *P* < 0.001). Moreover, Cox regression models were also constructed to further assess the survival difference. Both univariate and multivariate analyses indicated that the immunotherapy group had a significantly better clinical outcome compared to the no immunotherapy group (multivariate: HR = 0.75; 95% CI: [0.70–0.80]; *P* < 0.001) (Supplemental Digital Content Table 1, available at: http://links.lww.com/JS9/F209). Following propensity score matching at a 1:1 ratio, 3142 patients were paired according to their immunotherapy status. As shown in Table 1, no statistical difference was observed for all covariates after PSM with *P* values > 0.05. The Kaplan–Meier curve showed that patients who received immunotherapy had a better prognosis than those who had not (Fig. 2B). The multivariate Cox regression analyses further confirmed this survival benefit of immunotherapy (HR = 0.77; 95% CI: [0.70–0.85]; *P* < 0.001) (Table 3). Additional factors were identified to be associated with inferior survival, including older age (70+ vs age < 35; HR = 1.56; 95% CI: [1.15–2.12]; *P* = 0.004), black race (vs white; HR = 1.21; 95% CI: [1.05–1.39]; *P =* 0.008), brain (HR = 1.47; 95% CI: [1.22–1.78]; *P* < 0.001), liver (HR = 1.63; 95% CI: [1.45–1.83]; *P* < 0.001) and lung (HR = 1.23; 95% CI: [1.10–1.37]; *P* < 0.001) metastases, and received radiotherapy (HR = 1.15; 95% CI: [1.04–1.27]; *P =* 0.008). Conversely, undergoing hormone therapy (HR = 0.46; 95% CI: [0.41–0.53]; *P* < 0.001) and chemotherapy (HR = 0.62; 95% CI: [0.56–0.68]; *P* < 0.001) was associated with better outcomes.

**Table 3:** Univariate and multivariate Cox regression analysis for overall survival of *de novo* metastatic HR+HER2− breast cancer after PSM analysis

	Univariate analysis		Multivariate analysis	
Variables	HR (95% CI)	*P* value	HR (95% CI)	*P* value
Age		<0.001		<0.001
	Reference		Reference	
35–49	0.78 (0.57–1.06)	0.112	0.87 (0.64–1.20)	0.402
50–69	0.94 (0.70–1.26)	0.678	1.05 (0.78–1.42)	0.756
≥70	1.38 (1.02–1.86)	0.036	1.56 (1.15–2.12)	0.004
Race		<0.001		<0.001
White	Reference		Reference	
Black	1.23 (1.08–1.40)	0.002	1.21 (1.05–1.39)	0.008
Others/unknown	0.67 (0.53–0.84)	<0.001	0.72 (0.58–0.91)	0.005
Income		<0.001		<0.001
	Reference		Reference	
$38 000–47 999	0.94 (0.80–1.10)	0.453	1.05 (0.90–1.24)	0.524
$48 000–62 999	0.88 (0.75–1.02)	0.082	0.97 (0.83–1.14)	0.746
$63 000 or more	0.71 (0.61–0.82)	<0.001	0.79 (0.67–0.92)	0.002
Unknown	0.87 (0.73–1.03)	0.105	0.99 (0.84–1.19)	0.991
Location	–	0.670	NA	–
Metro	Reference			
Urban	1.01 (0.87–1.18)	0.870	–	–
Rural	1.19 (0.87–1.65)	0.281	–	–
Unknown	1.18 (0.91–1.54)	0.217		
Diagnose year		0.189	NA	
2013–2017	Reference			
2018–2021	0.94 (0.85–1.03)			
T stage	–	0.127	–	0.109
T1	Reference		Reference	
T2	1.03 (0.89–1.19)	0.704	1.03 (0.89–1.19)	0.694
T3	1.09 (0.93–1.29)	0.289	1.04 (0.89–1.23)	0.608
T4	1.16 (0.99–1.34)	0.051	1.16 (1.00–1.35)	0.046
N stage	–	0.368	NA	–
N0	Reference			
N1	1.09 (0.97–1.23)	0.148	–	–
N2	1.07 (0.90–1.28)	0.435	–	–
N3	1.14 (0.98–1.34)	0.098	–
Hormone therapy	–	<0.001	–	<0.001
No	Reference		Reference	
Yes	0.51 (0.45–0.57)		0.46 (0.41–0.53)
Chemotherapy	–	<0.001	–	<0.001
No	Reference		Reference	
Yes	0.68 (0.62–0.75)	–	0.62 (0.56–0.68)
Radiotherapy	–	0.045	–	0.008
No	Reference		Reference	
Yes	1.10 (1.02–1.21)		1.15 (1.04–1.27)
Metastatic site	–	–	–	–
Bone	1.03 (0.89–1.20)	0.648	NA	–
Brain	1.58 (1.32–1.89)	<0.001	1.47 (1.22–1.78)	<0.001
Liver	1.65 (1.48–1.85)	<0.001	1.63 (1.45–1.83)	<0.001
Lung	1.39 (1.25–1.55)	<0.001	1.23 (1.10–1.37)	<0.001
Immunotherapy		<0.001	–	<0.001
No	Reference		Reference	
Yes	0.83 (0.76–0.92)	–	0.77 (0.70–0.85)

CI, confidence interval; HR, hazard ratio.

**Figure 3.**
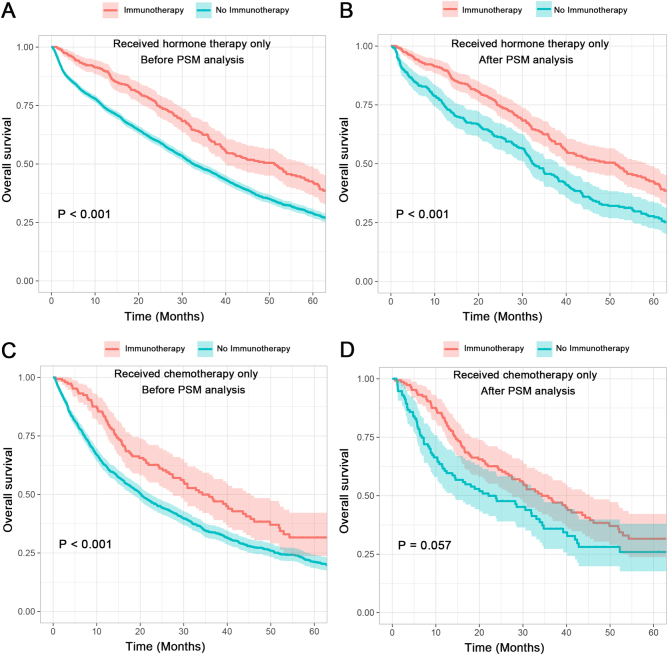
OS in subgroups with or without immunotherapy. (A) OS comparison for patients on hormone therapy only, pre-PSM; (B) OS comparison for patients on hormone therapy only, post-PSM; (C) OS comparison for patients on chemotherapy only, pre-PSM; and (D) OS comparison for patients on chemotherapy only, post-PSM.

Subsequently, the subgroups that only received hormone therapy or chemotherapy were selected and subjected to survival analysis. Figure 3A and B showed that receiving immunotherapy is significantly related to improved clinical outcomes of patients who had only received hormone therapy both before and after PSM (*P* < 0.05). The multivariate Cox analyses further indicated use of immunotherapy as a protective factor for such patients after PSM (HR = 0.62; 95% CI: [0.51–0.76]; *P* < 0.001) (Table 4). In terms of subgroups that only received chemotherapy, the Kaplan–Meier curve showed a relatively obvious trend of survival benefit (Fig. 3C and D). While the *P* values obtained from the univariate Cox analysis and the Kaplan–Meier method following PSM were close to significance, at 0.058 and 0.057 respectively (Fig. 3D and Table 4), the multivariate Cox analysis demonstrated a statistically significant association between immunotherapy and enhanced survival prognosis (HR = 0.67; 95% CI: [0.38–0.92]; *P* = 0.015).

**Table 4:** Cox analysis for overall survival among subgroups before and after PSM analysis

	Univariate analysis			Multivariate analysis	
	HR (95% CI)	*P* value		HR (95% CI)	*P* value
Before PSM					
Received hormone therapy only	0.68 (0.59–0.78)	<0.001		0.68 (0.58–0.78)	<0.001
Received chemotherapy only	0.68 (0.54–0.84)	<0.001		0.64 (0.51–0.80)	<0.001
After PSM	–	–	–	–	–
Received hormone therapy only	0.66 (0.55–0.80)	<0.001		0.62 (0.51–0.76)	<0.001
Received chemotherapy only	0.74 (0.54–1.01)	0.058		0.67 (0.48–0.92)	0.015

CI, confidence interval; HR, hazard ratio.

## Discussion

This large-scale NCDB analysis evaluated the survival advantage of immunotherapy for patients with newly diagnosed metastatic HR+/HER2− breast cancer. To our knowledge, this represents one of the largest analyses using real-world data to evaluate immunotherapy-associated survival outcomes specifically in metastatic HR+/HER2− breast cancer, providing important insights into a clinical scenario where evidence is limited.

Analysis of demographic and clinical characteristics revealed several patterns in immunotherapy utilization. Immunotherapy was less likely to be used in older patients, particularly those aged 70 and above, which may reflect concerns about potential toxicities associated with immunotherapeutic agents in elderly populations, who often have reduced physiological reserve and have more comorbidities. We observed racial and geographic disparities in immunotherapy access, with white patients and those living in metropolitan areas more likely to receive immunotherapy compared to black patients (OR = 0.83; *P* = 0.022) and those in urban areas (OR = 0.82; *P* = 0.022), respectively. These findings are consistent with previous studies examining racial and socioeconomic disparities in access to immunotherapy for other forms of metastatic cancer^[15,16]^. These disparities may reflect broader social and economic factors as well as differences in treatment approaches between academic medical centers and community hospitals. In addition, the cost factor may play a very important role in the application of immunotherapy use in daily practice, especially in low- and middle-income countries. To alleviate this issue, we think the following strategies could be attempted: (1) Support initiatives like drug donation programs or financial aid schemes; (2) Choose less expensive PD-L1 inhibitors based on clinical efficacy and patient affordability, while ensuring safety and efficacy; (3) Expand insurance coverage or implement price controls.

Notably, immunotherapy use was more frequent in patients with extensive lymph node involvement (N3 vs N0: OR = 1.35; *P* = 0.001), suggesting that clinicians may preferentially use advanced treatments in patients with poor prognostic features to achieve better disease control. Our data also revealed that treatments were important predictors of immunotherapy use, with positive associations observed between immunotherapy utilization and hormone therapy (OR = 1.21; *P* = 0.012) and radiotherapy (OR = 1.19; *P* = 0.002), which might indicate potential synergistic effects. Research has shown that several chemotherapeutic agents can enhance antitumor immune responses through dual mechanisms: triggering the release of immune-activating molecules from cancer cells undergoing cell death or exerting secondary effects on immune cell subpopulations^[17]^. Preliminary results suggest that combining immunotherapeutic agents with hormonal treatments may potentially boost therapeutic efficacy and clinical outcomes^[18]^. Moreover, poor response to endocrine therapy has been associated with increased TILs^[19]^. CDK4/6 inhibitors, used as the initial endocrine treatment for metastatic HR+/HER2− breast cancer, have demonstrated an ability to boost tumor immunogenicity and work in conjunction with PD-1 blockade to improve antitumor efficacy^[20,21]^. A growing body of evidence also supports the idea that radiotherapy can modulate immune responses, either enhancing or inhibiting the immune system through a variety of mechanisms^[22]^. These stimulatory effects of radiotherapy include the activation of antigen-presenting cells, the release of chemokines that recruit T cells, and the activation of the STING pathway.

HR+/HER2− breast cancer has historically been considered immunologically “cold” and characterized by low immunogenicity, likely due to the scarcity of TILs, the prevalence of tumor-associated macrophages, and diminished HLA class I expression, all of which hinder its antitumor immune response^[23]^. Nevertheless, a recent study examining TILs in ER +/HER2− breast cancer indicated that certain high-risk subgroups may exhibit greater immunogenic potential, warranting further investigation of immunotherapy strategies^[24]^. While the prognosis data is not yet mature, recent trials such as CheckMate-7FL and KEYNOTE-75 have demonstrated that the addition of neoadjuvant immunotherapy to chemotherapy significantly enhanced pathological complete response rates in early-stage high-risk HR+/HER2− breast cancer^[25,26]^. These results highlight the importance of better understanding the heterogeneity of HR+/HER2− breast cancer and identifying patient subgroups that are more likely to respond to immunotherapy. Currently, data on the efficacy of immunotherapy in metastatic HR+/HER2− breast cancer and long-term clinical outcomes are limited. Early trials such as KEYNOTE-028 and JAVELIN indicated limited clinical benefit of monotherapy ICIs, pembrolizumab and avelumab, in treating metastatic HR+/HER2− breast cancer^[7,8]^. In pursuit of enhancing efficacy through combination strategies, the KELLY trial reported promising antitumor responses with pembrolizumab administered alongside eribulin in patients previously exposed to multiple lines of therapy^[9]^. However, findings from the NCT03051659 study revealed no statistically significant improvement in PFS or objective response rate when comparing eribulin plus pembrolizumab to eribulin monotherapy^[27]^. The ongoing phase 3 KEYNOTE-B49 trial is assessing the impact of adding pembrolizumab to chemotherapy in metastatic HR+/HER2− breast patients with PD-L1+ (CPS ≥1) who have progressed following previous endocrine therapy^[28]^. However, as of now, the results of this investigation have not been disclosed. In our retrospective study, immunotherapy was linked to reduced mortality risk (HR 0.77, *P* < 0.001). For comparison, the NCDB data reveal an HR value of 0.59 for immunotherapy in advanced triple-negative breast cancer^[29]^. While the survival benefit appears more modest than in TNBC, it remains clinically relevant given the limited options in metastatic HR+/HER2− disease. The survival benefit from immunotherapy usage was also statistically significant in subgroups that received hormone therapy (HR: 0.62, *P* < 0.001) or chemotherapy (HR: 0.67, *P* = 0.015) only, which further indicates that ICIs have shown promise as an adjunct to established treatment strategies for advanced HR+/HER2− breast cancer.

Our findings may have significant implications for clinical decision-making in patients with metastatic HR+/HER2− breast cancer. This consistent survival benefit observed with immunotherapy across multiple analyses suggests that this treatment modality deserves consideration in the therapeutic algorithm for these patients, especially for those with high-risk features such as extensive lymph node involvement. Our results indicating potential synergy between immunotherapy and both hormone therapy and chemotherapy may guide combination treatment strategies. Furthermore, our findings regarding demographic and geographic disparities in immunotherapy access highlight the need for efforts to ensure equitable distribution of novel cancer therapies. The data we have collected might also aid in shaping future clinical trial designs for immunotherapy targeting metastatic HR+/HER2− breast cancer, particularly regarding combination strategies with established treatments and patient selection based on disease burden or other clinical characteristics.

Several limitations of our study warrant consideration. First, as an inherent limitation of retrospective database analyses using the NCDB, we lacked information on details of immunotherapy regimen, dosing schedules, combination strategies, and duration of treatment. Second, the NCDB does not include data on important biomarkers such as PD-L1 status, TMB, or genomic signatures. Lack of PD-L1/TMB data precludes biomarker-driven subgroup analysis, which is critical for optimizing patient selection. Third, we did not have access to information on PFS, treatment-related toxicities, treatment completion rates, tolerance, or quality of life outcomes, which are important considerations in evaluating the overall benefit of immunotherapy.

## Conclusion

In conclusion, our analysis of a large national database suggests that immunotherapy provides a significant survival benefit for metastatic HR+/HER2− breast cancer. The observed benefit was maintained across subgroups receiving different treatment modalities, suggesting potential versatility in combination strategies. These results address an important knowledge gap in breast cancer immunotherapy and have direct implications for clinical practice and upcoming research. Prospective clinical trials are warranted to further define optimal patient selection, timing, and combination strategies for immunotherapy in such types of breast cancer.

## Supplementary Material

## Data Availability

The data generated and/or analyzed during the current study are available in the NCDB database (https://www.facs.org/quality-programs/cancer-programs/national-cancer-database/). We confirm that all data-use agreements for the NCDB have been obtained and that the analysis complies with NCDB policies.
