# Supplementary material for: Favorable outcome of immunotherapy use in metastatic HR+/HER2− breast cancer: a population-based cohort study
Source: Int J Surg. 2025 Sep 23;112(1):1353–62. doi: 10.1097/JS9.0000000000003556 (PMC12825736; doi:10.1097/JS9.0000000000003556)

Supplementary Figure 1. Histogram of standardized differences before and after PSM. (A) Raw patients received immunotherapy; (B) Matched patients received immunotherapy; (C) Raw patients received no immunotherapy; (D) Matched patients received no immunotherapy.


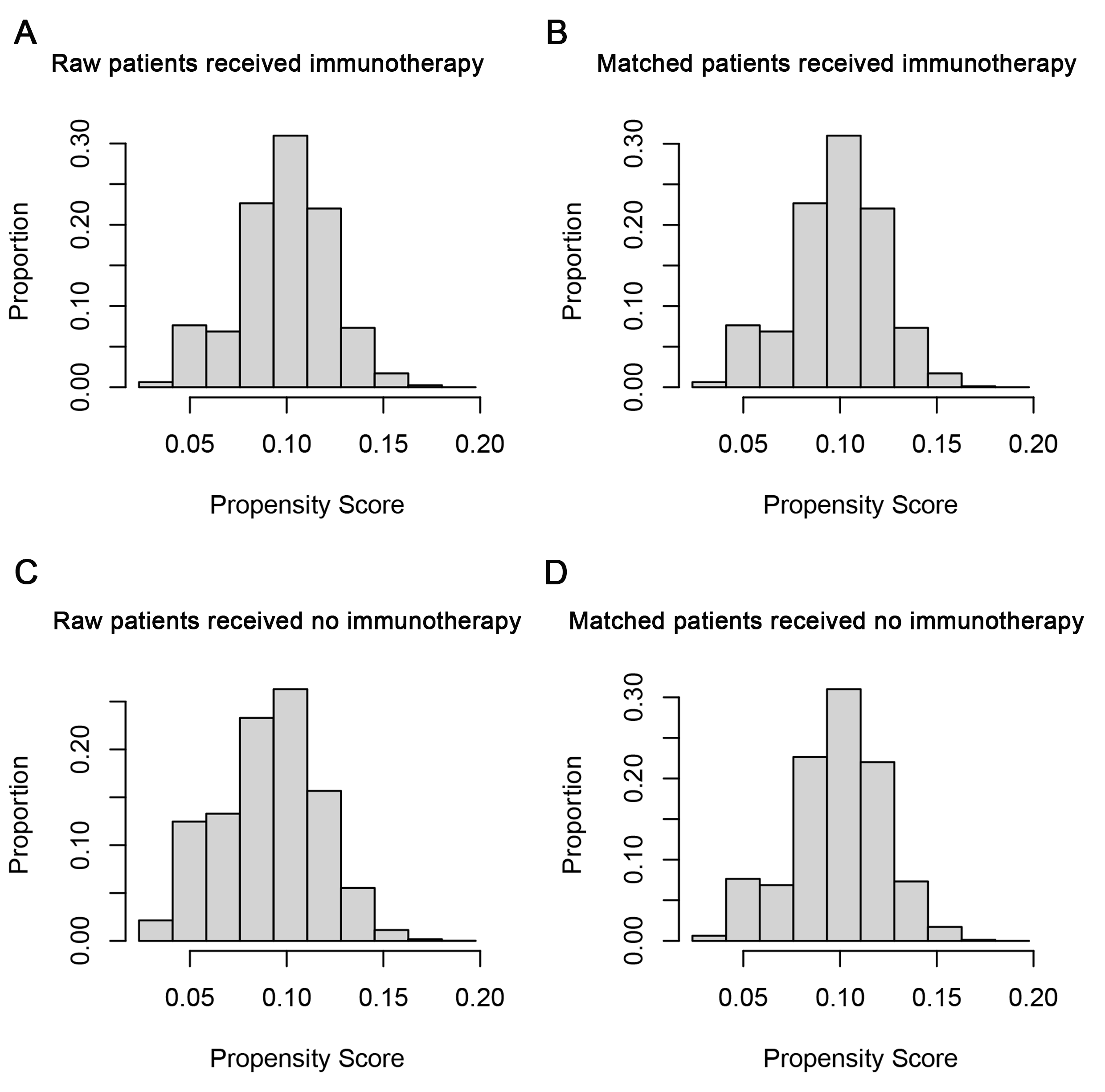

Supplement: Supplementary file 1 [file js9-112-1353-001.docx]
