# Supplementary material for: Favorable outcome of immunotherapy use in metastatic HR+/HER2− breast cancer: a population-based cohort study
Source: Int J Surg. 2025 Sep 23;112(1):1353–62. doi: 10.1097/JS9.0000000000003556 (PMC12825736; doi:10.1097/JS9.0000000000003556)
Supplement: Supplementary file 2 [file js9-112-1353-002.docx]

Supplementary Table 1. Univariate and Multivariate Cox regression analysis for overall survival of de novo metastatic HR+HER2- breast cancer before PSM analysis.

|  | Univariate analysis | |  | Multivariate analysis | |
| --- | --- | --- | --- | --- | --- |
| Variables | HR (95% CI) | P value |  | HR (95% CI) | P value |
| Age |  | <0.001 |  |  | <0.001 |
| <35 | Reference |  |  | Reference |  |
| 35-49 | 0.97 (0.84-1.12) | 0.711 |  | 0.95 (0.83-1.10) | 0.518 |
| 50-69 | 1.26 (1.10-1.45) | 0.001 |  | 1.31 (1.15-1.50) | <0.001 |
| ≥70 | 1.78 (1.56-2.04) | <0.001 |  | 1.80 (1.57-2.07) | <0.001 |
| Race |  | <0.001 |  |  | <0.001 |
| White | Reference |  |  | Reference |  |
| Black | 1.22 (1.15-1.28) | <0.001 |  | 1.17 (1.11-1.23) | <0.001 |
| Others/Unknown | 0.72 (0.66-0.79) | <0.001 |  | 0.76 (0.69-0.83) | <0.001 |
| Income |  | <0.001 |  |  | <0.001 |
| <$38000 | Reference |  |  | Reference |  |
| $38000-$47999 | 0.94 (0.88-1.00) | 0.066 |  | 1.00 (0.94-1.07) | 0.997 |
| $48000-$62999 | 0.90 (0.84-0.96) | 0.001 |  | 0.97 (0.91-1.04) | 0.383 |
| $63000 or more | 0.78 (0.73-0.82) | <0.001 |  | 0.85 (0.80-0.91) | <0.001 |
| Unknown | 0.87 (0.81-0.93) | <0.001 |  | 0.95 (0.89-1.03) | 0.199 |
| Location |  | <0.001 |  |  | 0.005 |
| Metro | Reference |  |  | Reference |  |
| Urban | 1.03 (0.97-1.09) | 0.371 |  | 0.98 (0.92-1.03) | 0.399 |
| Rural | 0.92 (0.79-1.06) | 0.236 |  | 0.98 (0.85-1.13) | 0.790 |
| Unknown | 0.73 (0.65-0.82) | <0.001 |  | 0.81 (0.72-0.91) | <0.001 |
| Diagnose year |  | <0.001 |  |  | 0.997 |
| 2013-2017 | Reference |  |  | Reference |  |
| 2018-2021 | 0.87 (0.84-0.90) |  |  | 1.00 (0.96-1.04) |  |
| T stage |  | <0.001 |  |  | <0.001 |
| T1 | Reference |  |  | Reference |  |
| T2 | 1.04 (0.98-1.10) | 0.201 |  | 1.08 (1.02-1.15) | 0.014 |
| T3 | 1.08 (1.01-1.15) | 0.035 |  | 1.10 (1.03-1.18) | 0.007 |
| T4 | 1.19 (1.12-1.26) | <0.001 |  | 1.18 (1.11-1.26) | <0.001 |
| N stage |  | 0.270 |  | NA |  |
| N0 | Reference |  |  |  |  |
| N1 | 0.99 (0.95-1.04) | 0.756 |  |  |  |
| N2 | 1.04 (0.97-1.11) | 0.264 |  |  |  |
| N3 | 1.03 (0.97-1.10) | 0.339 |  |  |  |
| Hormone therapy |  | <0.001 |  |  | <0.001 |
| No | Reference |  |  | Reference |  |
| Yes | 0.42 (0.40-0.44) |  |  | 0.43 (0.41-0.45) |  |
| Chemotherapy |  | <0.001 |  |  | <0.001 |
| No | Reference |  |  | Reference |  |
| Yes | 0.60 (0.57-0.62) |  |  | 0.60 (0.58-0.63) |  |
| Radiotherapy |  | 0.001 |  |  | <0.001 |
| No | Reference |  |  | Reference |  |
| Yes | 1.07 (1.03-1.12) |  |  | 1.14 (1.10-1.20) |  |
| Metastatic site |  |  |  |  |  |
| Bone | 0.98 (0.94-1.03) | 0.439 |  | NA |  |
| Brain | 1.93 (1.80-2.07) | <0.001 |  | 1.68 (1.56-1.81) | <0.001 |
| Liver | 1.76 (1.68-1.84) | <0.001 |  | 1.81 (1.72-1.89) | <0.001 |
| Lung | 1.28 (1.23-1.34) | <0.001 |  | 1.11 (1.06-1.16) | <0.001 |
| Immunotherapy |  | <0.001 |  |  | <0.001 |
| No | Reference |  |  | Reference |  |
| Yes | 0.74 (0.69-0.80) |  |  | 0.75 (0.70-0.80) |  |

Abbreviations:

CI: confidence interval; HR: Hazard ratio;
